# The care process of diabetic foot ulcer patients: a qualitative study in Iran

**DOI:** 10.1186/2251-6581-11-27

**Published:** 2012-12-19

**Authors:** Mansooreh Aliasgharpour, Nahid Dehghan Nayeri

**Affiliations:** 1 Department of Medical Surgical, School of Nursing and Midwifery, Tehran University of Medical Sciences, Tehran, Iran; 2 Nursing and Midwifery Care Research Center, School of Nursing and Midwifery, Tehran University of Medical Sciences, Tehran, Iran

**Keywords:** Care, Education, Diabetics, Qualitative study, Wound care

## Abstract

**Background:**

The Purpose of this study is to clarify the care process for Iranian diabetic patients with diabetic foot ulcer condition.

**Methods:**

The main question of this research was “How is the care process for diabetic foot ulcer patients and how do patients experience it?” This study was within the Grounded Theory method. Data collection was carried out until data saturation was achieved. Saturation was achieved after interviewing 11 patients, 4 physicians, one head nurse and one nurse.

**Results:**

Three main themes emerged from this study, including: “disease management, disease experience and continuity of care”. Each of these themes is consisted of different sub-themes.

**Conclusions:**

This is the first study to describe the care process in Iranian diabetic patients with diabetic foot ulcer disease. Knowing patients’ experience and the manner of dealing with them once faced with foot ulcer condition could facilitate a comprehensive decision making by therapists and better recovery of diabetic patients.

## Introduction

The disease of diabetes is one of the important problems of the world and the number of patients suffering from it, is growing day by day. Barathmanikanth declared the number of diabetics in the world to be 230 million people [1]. Diabetes challenges patients with numerous complications; even the proper treatment of diabetes, type 2, goes with cardio diseases, neuropathy, nephropathy, retinopathy and the diabetic foot syndrome [2]. In addition, more than 20 percent of patients, who suffer from the diabetic foot syndrome, experience amputation during their lives [3]. Unfortunately, the cost of diabetic foot ulcer has not been estimated precisely in Iran but expenses of patients with foot ulcer condition is very high and it decreases patients’ quality of life due to reduced mobility, causing disability and changing the mental image of patients about their body and pain [4]. Research has shown that behavioral factors are effective in bringing success for treating the diabetic foot ulcer disease but patients do not follow these recommendations thoroughly [5]. Meanwhile, the care procedure for diabetic foot ulcer patients is not truly known and patients’ experience is often ignored. Besides, few researches have been done about foot ulcer in Iran and most of them are quantitative studies which have emphasized on a specific aspect of the phenomenon and have failed to examine the whole process. It seems that identifying diabetic foot ulcer recuperation process and knowing patients’ experience could reduce this problem to some extent. In this regard, Vileikyte, et al., reported that being aware of patients’ knowledge about this condition and its treatment process may act as an aid in prevention and treatment of this disease [5,6]. Nevertheless, in order to clarify the aforementioned process, the researchers selected the qualitative research method. According to the researchers and by importance of the already mentioned points, the effective factors and obstacles of care process of foot ulcer condition can be known, through conducting more extensive research and gaining experience about this process. In addition, patients can be assisted in self-care and nurses in fulfilling the requirements of client’s and guaranteeing the caring quality. The present study was designed and implemented to clarify the care process for diabetic foot ulcer.

## Research design and methods

### Sample and setting

With regard to the main question of this research which was “How is the care process for the diabetic foot ulcer patients and how do patients experience it?” the grounded theory method was selected t for conducting this study. Grounded theory method (GT) is a systematic methodology in the social sciences involving the discovery of theory through the analysis of data. It is mainly used in qualitative research, but is also applicable to quantitative data ( [7,8]).

Grounded theory method is a research method which operates almost in a reverse fashion from traditional social science research. Rather than beginning with a hypothesis, the first step is data collection, through a variety of methods. From the data collected, the key points are marked with a series of codes, which are extracted from the text. The codes are grouped into similar concepts in order to make them more workable. From these concepts, categories are formed, which are the basis for the creation of a theory, or a reverse engineered hypothesis. This contradicts the traditional model of research, where the researcher chooses a theoretical framework, and only then applies this model to the phenomenon to be studied [9].

In this research, purposive sampling was done for patients with diabetic foot ulcer condition, which was followed by doing analysis and interviews simultaneously. After interviewing 11 patients suffering from foot ulcer disease who were hospitalized at one of the biggest government and university affiliated hospitals in the city of Tehran, data analysis directed us toward collecting data from nurses and physicians (6 individuals) to complement the process. Data collection continued until no new data could be added and data saturation was achieved. Overall, the number of participants reached 17 people. In order to collect the data, the demographic questionnaire and interviews, based on interview guideline, were used.

### Tools

The demographic questionnaire consisted of information such as age, gender, duration of suffering from the disease, record of using glibenclamide tablets, insulin usage record, having any record of spontaneous healing of wounds and amputation. The interview guideline was designed in the form of question: how did this wound occur? What did you do when you noticed your foot ulcer? What procedure do you adopt to take care of your foot ulcer? What kind of experience do you have in this regard that best describes your foot ulcer care? What obstacles have you faced in taking care of your foot ulcer and improving it? For benefit of the therapeutic team, the questions were as follows: what are your affaires about the diabetic patient? And what do you do when facing diabetic foot ulcer? What do all of your colleagues do in this regard?

### Procedure

After getting permission from university, hospital authorities and also the participants, the interview started and accordingly tape recorded. Written consents were obtained from the participants. The duration of each interview was between 30 to 45 minutes. After each interview, the recorded content was fully transcribed and was reviewed several times in order to achieve a total understanding of the interview and extract the hidden meanings in it. Then, the main themes were formed through negotiating ideas with team members. The previous topics were clarified more by continuing the interviews and sometimes new topics emerged. Throughout the conducted interviews with the patients, results led the researchers toward health care improvements and the implicit and explicit meanings were obtained from the data. 17 interviews were done in total during 9 months of the study. Field notes were taken at the same time as the interviews were conducted. Thus, when the researcher waited for the interviews to be finished or was referred to do the necessary coordination, she observed and examined the setting and the interactions. The manner of health care providers at the therapy center was noted and even some questions were asked from them by reference to these field notes. The temporary categories were formed by the progression of the research and merging of the codes in the first level.

### Ethical considerations

We followed all the principles for confidentiality of the data and getting the conscious consent for the interviews and tape-recordings. Having the right to stop participating in the study at any time was among the ethical principles that were followed.

## Findings

**Figure 1:**
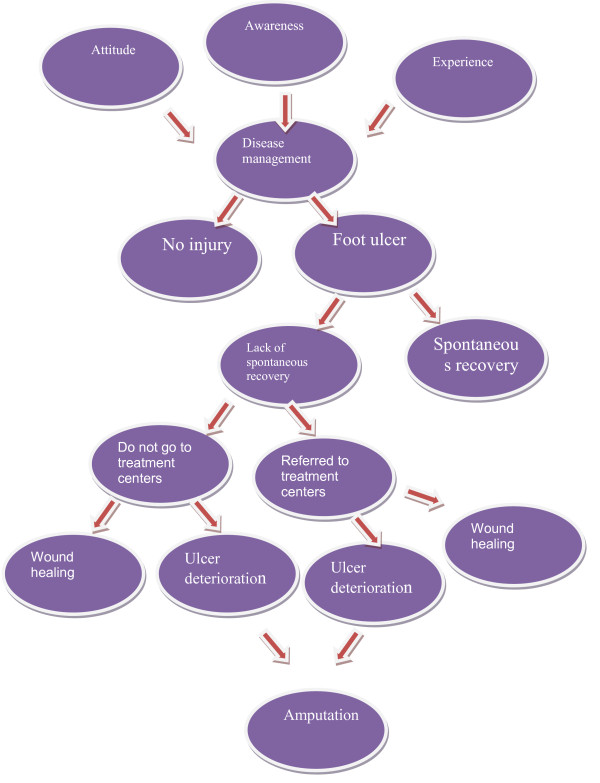
The relationship between experience, awareness, attitude, disease management and ulcer.

**Figure 2:**
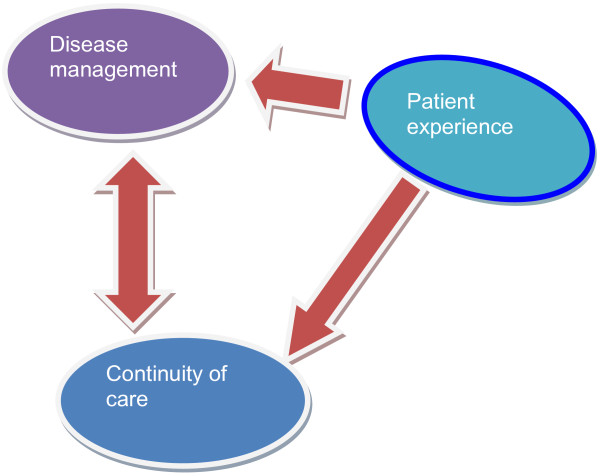
The relationship between continuity of care, disease management and patient experiences.

**Figure 3:**
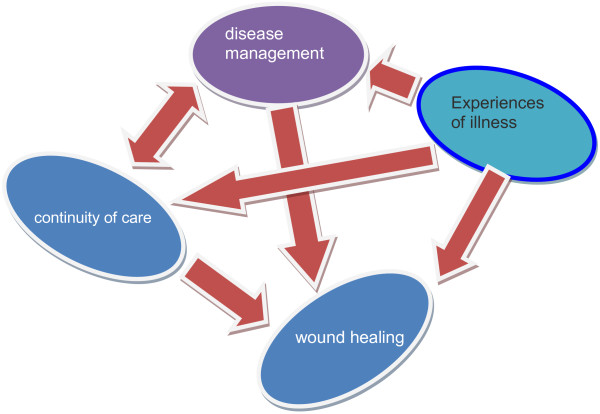
The relationship between experiences of illness, disease management, continuity of care with wound healing.

The participants in the present study included 17 individuals (11 diabetic patients suffering from foot ulcer and 6 physicians and nurses). The patients were those who were hospitalized at the Endocrinology, Surgery and Infection wards or were treated as outpatients at the Infection and Endocrinology clinics. All participants at hospitals affiliated with one of the universities of medical sciences were those who worked there. Most research units (%29.5) aged from 57 to 67 years old; among them, 58.8 percent were female and 82.4 were married. The first patient, who had the inclusion criteria, was selected after being introduced by the ward nurse after being referred to the Endocrinology ward. According to the results of the interviews, patients face with different factors during their experience and thus do a series of actions which could worsen or improve their disease management which is itself related to continuation of care. The findings indicated that the weak management of the disease leads to other diseases and vital complications like foot ulcer. They also showed that the strategies that patients adopt in regard to their foot ulcer are affected by their experiences, awareness and attitudes (Figure 1). The analysis of the findings in our research indicated that weak performance, lack of a precise screen over education, absence of team work, and lack of facilities lead to weak care techniques of diabetic patients suffering from foot ulcer. The analysis of findings also showed that the experience of the disease and its management are undeniably related to the continuity of care (Figure 2) and that disease management, disease experience and continuity of care are effective on the recuperation process of the wound (Figure 3). In other words, the patients who had appropriate awareness and information about their disease, attended training classes and had experiences about foot care and modern wound dressing methods, provided a better care for their foot ulcer condition. However, this issue was relative and sometimes patients were compelled to refer to therapeutic centers due to lack of training and not receiving follow-up care from health care providers, which had led to foot ulcer. As it is shown in the diagram, the experience of disease is related to the management of disease, continuity of care and recuperation of the wound. On the other hand, the management of disease is related to the continuity of care and recuperation of the wound. Besides, the continuity of care, which includes patients training, establishing specialized centers of caring for diabetic patients and follow-up by health care providers and the patient, affects on the management of the disease and wound recuperation. Through analyzing the data, three main themes emerged in this regard which included: “disease management, disease experience, and care continuity”. These three main themes, which formed the general meaning of care continuity, consist of several sub-themes. These cases alongside the quotations of the participants are presented in the following section.

### Disease management

One of the theoretical categories which emerged in this study was disease management. The findings of the research indicated that disease management plays a significant role in the care process of foot ulcer among diabetic patients. The sub-categories of disease management included: “awareness, attention, patient’s attitude and control”.

#### Lack of awareness

One of the main factors for patient’s awareness about the presence of the disease was having information about nutritional and medicinal diets, the normal level of blood sugar and awareness about the likelihood of having ulcer due to diabetes which forms the basis for the control of diabetes and preventing its side effects. It was shown in our findings that some diabetic patients were unaware of their disease up to appearance of an ulcer or its explicit symptoms; they also did not have sufficient information about their nutritious diet and were unaware of the normal level of blood sugar. In addition, most of them were unaware of the fact that they were more in risk of getting a foot ulcer when they have diabetes. In what follows, some samples of the statements form participants are presented.

One of the participants said that, “I do not know if I have diabetes I just went to doctor and he gave me a bunch of pills”.

One of the patients explained that, “when I knew that my blood sugar was 220 after having a test; I used to think to myself that it would remain at that level for 6 months”. Another patient said, “My blood sugar was 300–400 suddenly, it rose to 600”.

#### Lack of attention

Lack of attention consisted of inattentiveness of health care providers in training and patient’s inattention towards training which is among the problems of care so that some patients did not receive due attention from the therapeutic team. Similarly, a majority of therapists believed that patients did not pay attention to given trainings. One of the patients said, “Doctors told me that I must wash my foot; not to go somewhere full of broken glass, and to walk by slippers at home”. On the other hand, patients believed that they had not received enough training. one of the patients said that his doctor had told him that he must stick to his diet but had not told him what to eat. He stated that, “based on doctor’s advice there was not much left for me to choose from when it came to food. I was young and it was very difficult for me. It has been three years that I haven’t had any stew or gourmet food, I try to fill up with only bread”.

#### Patient’s attitude

The research findings here indicated that patients’ outlook plays an important role in managing the disease in different areas. Patients’ approach to following a balanced diet was not desirable and they were mostly unsuccessful in that area.. One patient said, “I don’t lose weight no matter what my diet is”. He stated that, “going on a diet is a difficult thing to do”. Other patients said that because we did not care about our eating habits, we could not control our blood sugar level. They admitted that they would not have had these problems if they had followed their diet.

Patients’ behavior towards following their medicinal diet was different so that through the interviews, they admitted to this fact that they had taken their oral medicine precisely and regularly. However, further examinations showed that despite consuming oral medicines for diabetes, in some cases the level of blood sugar was not controlled; hence, the doctor either increased the number of pills or advised the consumption of insulin. Still, some patients refused to take insulin because they considered insulin consumption as a symptom of their disease deterioration. One of the patients said, “I just regularly took my pills Then, my doctor said that I had to inject insulin but I was afraid of insulin So, I didn’t take it but I have taken my pills regularly. It has been 6 , 7 months that I’ve been told to take insulin but I am afraid to do so”.

Patients’ approach to taking care of their feet indicated that most of them did not apply any foot-care since they did not believe that their foot might get injured. One patient said : “I did not ever think that my foot might be injured some day since my two other brothers and sisters suffer from diabetes but their feet have not been affected”.

Among the other important points in disease management is controlling the blood sugar, but patients’ blood sugar level indicates that their blood sugar has not been under control. In response to this question that “did you use to control your blood sugar?” a patient said that, “Yes, my children control my blood sugar level, It is on a decent level, 200–250”. Most patients said, “My blood sugar was ok It was between 150 to 300”. Another patient said, “Since my blood sugar did not change for 6 months, I did not do my tests regularly”. Contrary to what the therapeutic team stated that blood sugar control is the main problem of diabetic patients, patients were unable to do it due to a number of reasons. Most patients did not do regular tests for controlling their blood sugar level and some of them that did control it, were unaware of its normal rate.

#### Self treatment

Among the other problems with regard to control, self-treatment by patients themselves is notice worthy. Findings of this research indicate that most patients had adopted self-treatment by using the AD ointment for curing foot cracks, rubbing olive oil on the ulcer. In case those were not effective they put the foot in cold salt water for healing the ulcer as was admitted by one the diabetic patients, using honey-therapy at home once having the ulcer, rinsing by serum and betadine solution by the patients or their family members and lack of timely referring to doctor, using vinegar by spraying it over the ulcer from the 15-centimeter distance and using grape juice, nettle and syrup for controlling the blood sugar. After willful usage of a cream, one of the patients said that, “I do not know its name; it is one of those ordinary creams. After using it, it caused infection, my toe blackened and the infection started to spread to other parts”. Another patient said, with regard to his experience of self care, that, “one of my friends who had diabetes told me to put my foot twice a day in cold salt water So, I did it for 20–25 days. Then, the surface of the wound became brownish. I went to the doctor and he introduced me to a surgeon for doing surgery on it”.

Unawareness, inattentiveness and lack of controlling the blood sugar level lead to the deterioration of ulcers and according to the findings, all of these factors result in the poor management of the disease.

### Disease experience

Disease experience, which includes the total experiences of patient about the factors causing the ulcer, the importance of ulcer and its associated diseases, also consists of subcategories like regret, discomfort and complaints by patients. The findings about the patients’ experience indicated that the factors that have led to foot ulcer are: wearing inappropriate and sharp-pointed shoes, the spontaneous appearance of ulcers as blisters or after accidently getting hit in the foot. Also, the research findings showed that the consequence of ulcer among most patients was toe amputation and also knee amputation in one patient. On the other hand, patients had experience of diseases like prior record of heart stroke, renal complication, bleeding eyes, loss of vision, losing sight in one eye and undergoing dialysis. The patients’ statements in this regard are presented in what follows:

“Because of wearing sharp-pointed shoes, I had to hit my foot against the wall to fit in; so, my foot got sore”. Another patient said, “I felt heated in my foot; my foot got sore spontaneously”. One of the patients said about amputations that, “my right toe was wounded 8 years ago; and got amputated then”. Another patient said that, “when my foot got wounded last year, I went to doctor. He examined me and told me that my blood sugar was 400. Now, my foot has been amputated from the knee”. Some patients had experienced re-hospitalized due to deterioration of their ulcers and lack of recuperation and were waiting for amputation. Findings about patients’ ulcer at hospital indicate that certain facilitating factors like using modern dressing had resulted in recovery of the ulcer and factors like early release from hospital before gaining complete recuperation had resulted in worsening their condition and even to amputation of toes or foot. A patient said about modern dressing:, “doctor told me that I’d better use modern, company-produced dressing. So, I accepted. They come every four days from the company to change my dressing; my wound has become much better. I’ll be discharged tomorrow”.

Patients did not have any information with regard to the diseases associated with the diabetes disease and had not received any training about it. One of the patients stated that, “once, my blood sugar reached 600, I had a stroke and got hospitalized”. Another patient said, “I was told I should undergo dialysis”. Patients had received certain care after having wounds and referring to hospital or had been referred to other therapeutic centers. Patients’ experiences in this regard indicated that they had complaints over different issues, including lengthening their hospitalization, lack of care at hospital despite timely hospitalization, losing toes due to getting hospitalized at irrelevant wards, long intervals between referring to hospitals and getting hospitalized, and getting released from hospitals after gaining partial recuperation and its deterioration after some days.

One of the patients was too upset due to getting hospitalized for one month and delay in his treatment:, “They have done nothing for me here”. Another patient said, “I was kept in the hospital for 15 days at the emergency ward; my toe had completely blackened. They sent me to the operation room and amputated my toe. Then, I was transferred to the surgery ward and it has been 10 to 15 nights that I am still here”. Besides, our findings indicated that patients expressed feelings of regret and discomfort for not going through follow ups and on time referring to the therapeutic team. some of them were upset about the changes in their limbs. Subsequently, one of them said, “I am very upset about being disfigured in my foot”. Another patient said, “If I had come sooner, it would have been better to prevent it. My family and I got baffled when I was told that my foot needed to be amputated; I didn’t come in time and it was a mistake. Still, another one said, “how could I know the possibility of facing such miseries?” He continued by saying, “if I have controlled it, this would have been different”.

Patients’ experiences indicate that the consequences of this disease and those associated with it have not led to an appropriate follow-up from the side of the patient for taking care of the ulcer and have led to the referral of the patient for a second time to therapeutic centers.

### Central variable: continuity of care

Continuity of care is one of the main categories and it is actually the principal variable of the emerged theory out of the data. All other categories gather around this principal variable through care process for ulcers. This concept was the most obvious one that developed out of the data and it was the most abstract one that could cover all other categories and connect them to each other. Disease management and patient’s experiences indicate that the main issue for these patients is hidden in care process and its continuity. This principal variable consists of the subcategories of the therapeutic team’s performance, team work and deficiencies.

#### Therapeutic team’s performance

The research findings showed that the therapeutic team is mostly engaged in routine works and has ignored its main responsibility, which is offering training to patients and hence they do not pay due attention to patients. Moreover, the findings indicated that informing and providing services to patients are not done equally and even the weak informing that had been done was carried out after the occurrence of the ulcer. Among other findings in this regard was the weak supervision over the performance of therapeutic team by training supervisors. The statements of participants about this issue are presented in the following section.

One of the patients said, “The doctors working at the hospitals affiliated with the insurance company do not pay attention to us; they just repeat the previous prescriptions”. Another patient said, “The information was provided by therapeutic team after the occurrence of the ulcer”. Still, another patient said, “I was advised to attend classes after they prescribed insulin for me. Some team members told me that the introduction to the Diabetic Association or training classes is not done for all patients or that, training the diabetic patients is partial and a small number of patients receive these services”. Some of the patients even believed that, “sharing information about the existence of the diabetic association is achieved on the basis of patient’s willingness and follow-ups”. The findings indicate that patients’ participation or lack of participation at training classes are not attended to in therapeutic centers. After several days of referring to the centers, the researcher herself also noticed that only the patients hospitalized at the Endocrinology ward are provided with services because this ward has been considered as the center of diabetic patients, although the provided training is not enough and followed up afterwards. This issue indicates the weak administration of supervisors and the therapeutic team, since diabetic patients are hospitalized at other wards and do not receive these trainings.

#### Therapeutic team’s approach

Another point that plays a role in the continuity of care is the outlook of therapeutic team. Findings about their approach to this issue indicate that they point out the consequences of the disease to patients themselves. The complications resulting from the disease that inflict the patients are due to overlooking the disease and ignoring the therapeutic process since the main role of the team members is the treatment of the patients. The team believed that “patients do not have information about their disease or their information is insufficient”. They also believed that “patients are unaware of ways to prevent or examine the presence of senses at feet. They do not take care of their feet and also do not control their blood sugar level”. The attitude of some of team members was that “the main source of problem is the patient”.

Some team members believed that the required work is not done by the team and this causes problem. They also considered lack of a specialist team for curing the patients with foot ulcer as one of the biggest problems. Their statements are presented in the following section.

“The patient is not considered as a whole. One of the problems is lack of cooperation and sympathy among the specialists who are engaged in curing the diabetic patients with foot ulcer”. They also stated that, “the doctors, giving advice about curing the diabetic patients are not enough”. One of the team members believed that, “giving advice is done by the residents and the advising doctors do not pursue the follow ups for the patients”. In general, “they expressed lack of shared discussions among the specialists about the method of curing the patient as a fundamental problem”. They also admitted that, “there are certain problems in the process of treating diabetic patients and in order to treat their ulcers, group work must be followed up”.

Shortage of human resources and specialist centers was another influential matter in care process for the diabetic foot ulcer.

#### Shortage of human resources

One of the negatively effective shortages that were found through our explorations was the shortage of human resources. The majority of the team members stated that, “lack of availability to a trained individual and a nurse specializing in curing ulcers are among the main reasons for lack of success in curing the diabetic patients”.

Shortage of specialist centers: another shortage was the lack of specialist centers for curing the diabetic patients. The doctors in the therapeutic team considered having specialist clinics for foot ulcer disease as crucial and some of them believed that, “in case of having no specialist clinic for the diabetic patients, doctors do not see themselves as responsible”.

## Discussion

Patients’ unawareness about the presence of diabetic disease, disease process, the normal level of blood sugar, the effect of medicinal diet, type of nutritional diet, care process for the foot and preventing the ulcer [10,11] the likelihood for the occurrence of ulcer following the diabetic disease, presence of the diabetic association, and access to training classes results in the weak management of the disease and accordingly in the deterioration of the foot ulcer. Jaffiol also believes that the rate of amputation among the patients who suffer from the diabetic foot ulcer syndrome and have a weak control over their blood sugar is much higher [12]. This issue was also corroborated by our therapists who believed that the diabetic patients who suffer from foot ulcer condition do not control their blood sugar level and this indicated that patients do not get proper training in this regard. Meanwhile, other researchers have concluded that in order to prevent the complications following the diabetic disease, the therapists must offer training to patients since blood sugar control prevents the occurrence of these complications or at least postpones their occurrence. Lippmann-Grob also believed that systematic interventions are effective for controlling the blood sugar and if they are done at initial stages of the diabetic disease, they will be more effective for preventing complications [13]. This is also true about following a proper nourishing diet that patients usually do not follow due to excessive hunger or feeling good about the results of the tests and having a blood sugar of 200. Besides, some patients do not know that they must follow a special nutritional diet. The findings of the research conducted by Albine Moser were in contrast with our findings. In his research, participants had a good control over their blood sugar and they controlled their nutritional diet according to their blood sugar rate; they were being trained under the supervision of the diabetic specialist and could ask their questions so that they could have a better care of themselves [14]. In contrast, our patients quite accidentally knew of their disease following its signs and symptoms and had not done a proper caring themselves due to lack of awareness about the process of diabetes. The patients did not attend to their sore ulcer and to themselves at the initial stages of the ulcer and had sufficed on their own previous experiences and had practiced self-treatment. With the expansion of infection symptoms, bleeding and ulcer deterioration, the patient referred to the therapeutic team. The manner of dealing with the patient by the team was different so that some team-therapy centers treated patients as outpatients, while others hospitalized patients for a long time and had kept him waiting or under treated t. Some had referred the patient to bigger centers; while some had hospitalized the patients at non-specialist centers; still, some of them had opted for the amputation of the foot or the toes and others had released the patient after gaining partial recuperation which had resulted in the worsening his condition. Some researchers [15,16] emphasize on the diabetic patients training. Contrary to Lone Gale, who believes that the hygienic specialists must pursue their training to the extent that they would be assured that patients believe in these trainings [17], our therapeutic team was unable to do this and there was no systematic program for training the diabetic patients or they were very weak?

The therapeutic team is quite aware of the patients’ unawareness and primarily blames the patient as the main responsible person for the disease complications and points out very weakly to their own training duty. They consider training as influential but are unable to pursue a continuous training for the patients due to certain reasons including :lack of dividing the responsibilities at hospital, excessive workload, high number of patients, low number of therapeutic personnel, lack of patient training once diagnosing the disease, lack of facilities like the absence or shortage of instructional booklets or pamphlets, lack of trained nurses for footsore, absence of wound clinics and lack of real cooperation between medical advisors and specialists and not considering the patient as a whole by the therapeutic team. Rabi states that lack of access to instructional and advisory centers and specialist clinics and caring for patient by one physician and also lack of attention to hygiene instruction by the physician or others are among the factors that bring deficiency for providing care to the diabetic patients and must be attended to [18]. Hellar, who had also reached the same conclusions in his research about the diabetic patients, suggests that it is necessary to have a distinct therapeutic protocol for diabetic patients and it must be prepared by the therapeutic team members, including o surgeon, physicians, anesthesia specialists, physiotherapists, nutritional expert and nurses [19]. The diabetic patients must be referred to special centers for the diabetic foot and be hospitalized at specialized wards for diabetics, not at the internal-surgery or other wards. Rabi also believes that the presence of hygiene specialists at academic level is quite mandatory for giving the daily care trainings in order to prevent the diabetic foot syndrome [18].

It seems that the therapeutic team is being entangled in daily routines and does not have a systematic plan for training and curing the diabetic patients. Also, the analysis of the findings shows that the therapeutic team does not pay proper attention to this process because of a certain attitude toward the patient and blaming him/her for the occurrence of diabetes or foot ulcer-related complication; hence, they bring about a deficient cycle by avoiding to train the patients or giving them the required information about their disease. Thus, patients refer again and again to diabetic clinics or hospitals. The care process for the diabetic foot ulcer has resulted in satisfaction among some patients who had experienced the recuperation of their ulcer by using modern bandaging methods, while it had brought dissatisfaction for the patients who had experienced lengthy hospitalization, untimely consideration, long procedure of hospitalization, getting released after gaining partial recuperation and being re-hospitalized after deterioration of their condition. Studies [6,16,18] have also indicated that patients were not satisfied with the hygiene caretakers. Besides, Hellar indicates that patients get hospitalized at ward for a long time which is related to the higher number of patients compared to the number of nurses [19]. Pendsey mentions that, “It is quite obvious that caring for the diabetic foot requires examining the foot, checking the wound, its intensity and applying proper therapeutic techniques and it is clear that all of these affairs require a robust therapeutic team to train patients step by step for preventing his/her diabetic foot ulcer” [3].

The significance of team work in treatment and providing care for patients has been indicated in several papers [2,3,20]. Our therapeutic team is also aware of that and considers lack of team work as a big problem for treating the diabetic patients. However, the issue of team work in treating the diabetic patients is unfortunately very insignificant in our country and this makes the problem more complicated. Team work requires trained personnel. Shortages and lack of facilities are among the influential issues on the caring process for the diabetic foot ulcer. The findings of Agarwal, et al. in 2002 indicated that the main obstacles for providing care to diabetic patients are the shortage of human resources, having insufficient time for training and getting information about new protocols, excessive workload and lack of standardized protocols for treating the diabetic patients [21]. Among the findings of the present study, one of the influential deficiencies is human resources shortage. The majority of the therapeutic team members stated that the absence of a trained nurse or individual specializing in ulcer is among the main reasons for lack of success in treating the diabetic patients. The low number of nutritional experts and not allocating enough time for training diabetic patients what to eat and avoid were among other shortcomings.

## Conclusion

As it was said, various reasons contribute to recuperation of the diabetic foot ulcer. The most important ones are the disease experiences and disease management and also the continuity of caring. Attending to the latter point and having team therapy with a systematic program and continuous training for the patients alongside establishing specialist care centers for diabetic patients and employing trained nurses with specialty in foot diseases lead to the proper management of the disease and further participation from patients which consequently play a significant role in the recuperation of the foot ulcer syndrome.

## Competing interests

The authors declare that they have no conflicts of interests.

## Authors’ contributions

MA carried out the design and coordinated the study, participated in most of the experiments and prepared the manuscript. NDN provide assistance in the design of the study, coordinated and carried out all the experiments and participated in manuscript preparation. All authors have read and approved the content of the manuscript.
